# Trajectories of antidepressant use and 6-year change in body weight: a prospective population-based cohort study

**DOI:** 10.3389/fpsyt.2024.1464898

**Published:** 2024-12-24

**Authors:** Camille Lassale, Gabriela Lugon, Álvaro Hernáez, Philipp Frank, Jaume Marrugat, Rafael Ramos, Josep Garre-Olmo, Roberto Elosua

**Affiliations:** ^1^ Hospital del Mar Medical Research Institute (IMIM), Barcelona, Spain; ^2^ Consortium for Biomedical Research - Pathophysiology of Obesity and Nutrition (CIBEROBN), Instituto de Salud Carlos III, Madrid, Spain; ^3^ ISGlobal, Barcelona, Spain; ^4^ Universitat Pompeu Fabra (UPF), Barcelona, Spain; ^5^ Centre for Fertility and Health, Norwegian Institute of Public Health, Oslo, Norway; ^6^ Facultat de Ciènces de la Salut Blanquerna, Universitat Ramon Llull, Barcelona, Spain; ^7^ Research Department of Epidemiology and Public Health, University College London, London, United Kingdom; ^8^ Consortium for Biomedical Research - Cardiovascular Diseases (CIBERCV), Instituto de Salud Carlos III, Madrid, Spain; ^9^ Institut Universitari d’Investigació en Atenció Primària Jordi Gol (IDIAP Jordi Gol), Girona, Spain; ^10^ Girona Biomedical Research Institute (IdIBGi), Dr. Josep Trueta University Hospital, Girona, Spain; ^11^ Department of Medical Sciences, School of Medicine, University of Girona, Girona, Spain; ^12^ Department of Nursing, University of Girona, Girona, Spain; ^13^ Faculty of Medicine, University of Vic - Central University of Catalunya, Vic, Spain

**Keywords:** antidepressant, weight change, anthropometrics, obesity, prospective study

## Abstract

**Background:**

Antidepressant drug treatment may be associated with weight gain, but long-term studies are lacking.

**Methods:**

We included 3,127 adults (1,701 women) from the REGICOR study, aged 55.6 (SD = 11.6) years on average in 2003–2006, living in the northeast of Spain. They had data at two time points (baseline and a median of 6.3 years later) on self-reported antidepressant use, body weight and height, and on baseline smoking, physical activity, diet quality, education, civil status, and depressive symptoms assessed with the Patient Health Questionnaire (PHQ-9) at follow-up. We defined four trajectories of antidepressant use as follows: never use, new use at follow-up, initial use discontinued, repeated use at both time points. We used multivariable linear models to estimate the association of these trajectories with the percentage of weight change. In people without obesity at baseline (n = 2,404), we also estimated the association with obesity incidence at follow-up.

**Results:**

The average 6-year weight gain was 0.53 kg (1.01% body weight), and 24.5% of the participants gained >5% of body weight. The majority (83.6%) of participants did not report any use of antidepressants, 6.2% initiated during follow-up, 5.1% discontinued it, and 5.1% reported their use at both time points. In multivariable analyses, compared to never users, all trajectories were associated with greater weight gain: +1.78% (0.57, 2.98) for initial use discontinued, +2.08% (0.97, 3.19) for new use at follow-up, and +1.98% (95% CI: 0.75, 3.20) for repeated use. In non-obese participants at baseline (n = 2,404), the odds ratio for becoming obese was 2.06 (1.03, 3.96) for repeated use and non-statistically significant for the other trajectories.

**Conclusions:**

In a population-based adult cohort, repeated use of antidepressants was strongly associated with weight gain. New and discontinued use was associated with weight gain, but non-significantly to obesity incidence. Given the global obesity epidemic and the widespread use of antidepressants, weight management and metabolic monitoring should be encouraged and integrated into depression follow-up guidelines alongside antidepressant prescriptions.

## Introduction

The most prevalent psychiatric disorder is major depressive disorder (1). It is estimated that depression affects 280 million people globally (2) representing a prevalence of 5% among individuals aged over 20. Across the life course, women are nearly twice as likely to experience depression as men. Depression can have debilitating consequences ranking among the top two causes of years lived with disability (1). The main recommended treatment for major depressive disorder, in conjunction with psychological therapies, is antidepressant drugs (3, 4)—the most commonly prescribed class of medication worldwide. The prescription of an antidepressant contemplates both short (acute and continuation phase)- and long-term (maintenance phase) aspects, ranging from 1 year to lifelong (5). Data from the UK’s largest primary care database show that 24% of patients were prescribed antidepressants at least once between 1995 and 2011, more commonly in women than in men, and that the prevalence more than doubled in this period (3). In Spain (Castile and León region), a recent study reports 8.6% of annual users of antidepressants, with prevalence rates of 12.1% in women and 4.9% in men (6).

Despite improvements in the acceptability and tolerability of antidepressant drugs, they all have adverse effects, both at short and long term, and balancing benefits and harms is an ongoing concern (7). Listed side effects include insomnia, sleepiness, dizziness, restlessness, muscle spasms, dry mouth, profuse sweating, sexual disorders, nausea, constipation, diarrhea, and importantly weight changes (5). Weight gain has been shown to be an important reason for discontinuing treatment (8).

Obesity affects approximately 650 million people worldwide and has widespread health ramifications (9). Robust epidemiological evidence suggests a relationship between obesity and depression, likely bidirectional: longitudinal studies show that obesity is associated with higher incident depression and specific depressive symptoms (10), and that depression is associated with a higher risk of subsequent obesity (10–13). Several shared biological mechanisms have been proposed to explain this relationship, including genetics, hypothalamic–pituitary–adrenal axis hyperactivation, leptin dysregulation, insulin resistance, and gut dysbiosis (13). Another important candidate in explaining the association between depression and obesity is the use of antidepressants, which can produce weight gain as a side effect, although this varies by drug class. For example, some selective serotonin reuptake inhibitors (SSRIs) or tricyclic antidepressants (TCAs) have been associated with weight gain, whereas others, such as bupropion, demonstrate weight loss effects (14, 15). However, most of the evidence comes from short-term trials (weeks to months) (8, 16), and real-life longitudinal studies evaluating the effects of prolonged antidepressant use over the long term (years) are lacking (16, 17). The largest study to date used primary care data from the UK with a 10-year follow-up and showed that antidepressant use between 1 and 6 years was associated with higher risk of gaining more than 5% of body weight (18). A study in Finnish adults showed that prolonged use of any antidepressant (>200 daily doses/year for 4 years) was associated with a greater 4-year weight gain compared with equally depressed control individuals who had no record of antidepressant use (19). A Canadian study found that SSRIs and venlafaxine, but not TCAs, were associated with increased risk of becoming obese after 10 years (20). In a smaller study, only SSRI use was associated with a larger increase in BMI compared to nonusers, but not TCAs nor other antidepressants, in particular, when use lasted more than 90 days (21). To our knowledge, all studies have considered cumulative exposure, but none described long-term trajectories such as discontinued use of antidepressants.

Our aim was to describe trajectories of antidepressant use, 6 years apart, and their association with weight change and risk of obesity in a population-based longitudinal cohort study of Spanish middle-aged adults.

## Methods

### Study design and population

The REGICOR (REgistre GIroni del COR, or Girona Heart Registry) study is a prospective population-based cohort in Girona province (approximately 700,000 inhabitants) in northeastern Spain, with the primary objective of studying cardiovascular disease incidence and its risk factors. The study design has been described in detail elsewhere (22). Briefly, individuals living in 42 communities, including 41 villages and the city of Girona, were randomly selected from the census and invited to participate. Inclusion criteria required that participants aged 35–79 years were free of terminal disease, not institutionalized, and had lived in the referral area for at least 6 months per year. The recruitment took place in three consecutive periods as follows: 1995, 2000, and 2005. Of those invited, there was a 73.8% participation rate at the first visit. In this study, we used as a baseline only the sample of 6,352 participants from the third wave of recruitment (2005). The follow-up visit took place between 2007 and 2013, in which 2,072 participants did not attend. There was no visit in the interim, and the average difference between baseline and follow-up visit was 6 years. Out of the people who were eligible to come to the follow-up visit (alive, still living in the catching area, no serious disease), 78% participated in it. The study protocol was approved by the Parc de Salut Mar Research Ethics Committee, and each participant signed an informed consent form at enrollment. Examinations were performed by trained nurses and interviewers using standardized and validated questionnaires and measurement methods, as previously described (20).

### Exposure assessment

At baseline, medication use was self-reported by the participant in an open list. We considered as antidepressants those reclassified as SSRI, norepinephrine and dopamine reuptake inhibitors (NDRIs), and TCA. At follow-up, a specific question on psychopharmacological medication was filled by the researcher and classified as follows: antiepileptic, antiparkinsonian, antipsychotic, lithium, anxiolytics, hypnotics and sedative drugs, SSRIs, NDRIS, and other psychopharmacological drugs. We classified as antidepressants SSRIs and NDRIs.

We created a four-category variable to reflect the 6-year trajectory of use of antidepressants between baseline and the follow-up visit: never use, initial use discontinued (use at baseline but not at follow-up), new use at follow-up (no use at baseline, and use at follow-up), and repeated use (use at both baseline and follow-up). We also created a variable for the trajectory of SSRI use, as it was consistently reported at baseline and follow-up with adequate power to compute the four-category variable.

### Outcome

Body height and weight were measured by trained nurses at both visits using standard protocols. We computed body mass index (BMI) as the weight in kilograms divided by squared height in meters and defined obesity as having a BMI ≥30 kg/m^2^ (23). The relative change in body weight was defined as the difference between weight at follow-up minus weight at baseline, divided by weight at baseline, expressed as percentage.

### Covariates

All covariables were assessed at baseline, except depressive symptoms and civil status, which were reported at the follow-up visit.

Smoking (current, ex-, never smoker), civil status (married/cohabiting, not living with a partner), and educational level (low, medium, and high) were self-reported on standard questionnaires. Physical activity was assessed by the Minnesota Leisure Time Physical Activity Questionnaire validated for the Spanish population (24, 25). Dietary intake was assessed by a 157-item food frequency questionnaire (26), and we used the relative Mediterranean diet score ranging 0–18 (27) to assess diet quality. Type-2 diabetes was defined as presenting fasting glucose levels >126 mg/dl or being treated with insulin or glucose-lowering medications. Hypertension was defined as systolic blood pressure >140 mmHg, diastolic blood pressure >90 mmHg, or being a user of antihypertensive medication. Diabetes and hypertension can be both related to adiposity, change in behavior, and depression. Antipsychotic medication can induce weight gain, and individuals with depression might be concurrently treated with both antidepressants and antipsychotics. Therefore, we also considered antipsychotic use as a confounding factor. However, only 39 participants declared taking antipsychotics at baseline, and 34 at follow-up; therefore, the confounding was minimal.

Depressive symptoms were ascertained from the Patient Health Questionnaire (PHQ-9) validated in Spanish (28). This self-report measure sums the scores from 0 (“not at all”) to 3 (“nearly every day”) for a set of nine questions such as “Little interest or pleasure in doing things,” “Trouble falling asleep, staying asleep, or sleeping too much,” or “Feeling bad about yourself— or that you’re a failure or have let yourself or your family down.” The total score ranges from 0 to 27, and a score ≥10 defines presence of depression (29). Moreover, a linkage with primary care electronic health records (Programa d’analítica de dades per a la recerca i la innovació en salut -PADRIS-) of the Catalan Government was performed from 1 January 2008 up to 31 December 2016. We included in the definition of case of depression any of the two following ICD-10 codes (30): F32 (depressive episode) and F33 (recurrent major depressive disorder), and only included diagnosis that occurred between the baseline and follow-up visit. We created a composite outcome variable “depression” as having elevated depressive symptoms or depression during the follow-up. This was used as a key confounder to adjust for to disentangle the potential effect of antidepressant drugs from the effect of depression on body weight change.

### Statistical analysis

Description of baseline characteristics was performed by stratifying participants according to antidepressant trajectories and also to age group, sex, and baseline BMI category. Differences were assessed by ANOVA (continuous variables) or chi-square tests (categorical variables). Results are expressed as means (standard deviation) or numbers (%).

We modeled percent body weight change as a continuous outcome in generalized linear models, where antidepressant trajectory was the exposure categorical variable using “never use” as the reference category. Model 0 only included age and sex. Model 1 included age, sex, educational level, civil status, smoking, Mediterranean diet score, physical activity, diabetes, and hypertension as covariables. Age, Mediterranean diet score, and physical activity level were treated as continuous variables; sex, education, civil status, smoking, hypertension, and diabetes as categorical variables. Model 2 further included depression status aiming to disentangle depression from antidepressant use. Model 3 further adjusted for baseline BMI. In sensitivity analysis, we further adjusted for antipsychotic use. We further looked for interactions between use of antidepressants and age, sex, and baseline BMI that we formally tested with likelihood ratio tests of nested models with and without the interaction term and reported stratified results. Results are expressed as beta coefficients and 95% confidence intervals.

We also modeled the odds of weight gain >5%, which is a clinically relevant outcome and can make results comparable with a recently published study (18) by performing multivariable logistic regressions following the same adjustment strategy as above. Finally, after exclusion of participants with obesity at baseline, we assessed the odds of incident obesity at follow-up using the same modeling approach. Results are expressed as odds ratios and 95% confidence intervals.

Two-tailed p-values of less than 0.05 were considered statistically significant. All analyses were performed using R version 4.2.1.

## Results

A total of 3,127 adults (1,701 women), aged on average 55.6 years (SD = 11.6), were included in the main analysis sample with complete data (Flow chart **Supplementary Figure S1**). Compared with participants who were excluded from the present analysis (**Supplementary Table S1**), those included were younger, more likely to be female, never smoked, and were less likely to have low education. However, they did not differ significantly in their diet quality, physical activity levels, baseline BMI, and use of antidepressants.

The median follow-up was 6.3 years later (interquartile range 5.7–6.8 years, fifth percentile: 5.3 years, 95% percentile 9.9 years). Overall, 16.4% of the participants used antidepressant at either time point: 5.1% at baseline but discontinued, 6.2% initiated at follow-up, and 5.1% at both time points. The mean weight change over follow-up was +0.53 (SD = 6.19) kg, corresponding to +1.01 (7.88)% of initial body weight. Participant characteristics are presented in **Table 1**. Compared to never users, ever users (any other trajectory) were older, more likely to be female, non-smoker, less likely to have high educational level, to live with a partner, and repeated users presented lower physical activity levels and Mediterranean diet adherence score. Antidepressant ever users also presented more depressive symptoms, higher baseline and follow-up BMI, higher obesity prevalence, as well as greater weight gain.

**Table 1:** Characteristics of participants in the REGICOR study included in the analysis, overall and across antidepressant use trajectories (N=3,127), Spain, 2005-2013.

	Overall	Never use	Initial use discontinued	New use at follow-up	Repeated use	p-Value^a^
n (%)	3,127	2,613 (83.6)	161 (5.1)	194 (6.2)	159 (5.1)	
Female (%)	1,701 (54.4)	1,313 (50.2)	113 (70.2)	148 (76.3)	127 (79.9)	<0.001
Age [mean (SD)]	55.56 (11.61)	55.10 (11.67)	56.51 (10.79)	57.82 (11.42)	59.32 (10.53)	<0.001
Educational level (%)						0.003
High	715 (22.9)	628 (24.0)	30 (18.6)	31 (16.0)	26 (16.4)	
Low	1,486 (47.5)	1,209 (46.3)	77 (47.8)	106 (54.6)	94 (59.1)	
Medium	926 (29.6)	776 (29.7)	54 (33.5)	57 (29.4)	39 (24.5)	
Living with a partner (%)	2,369 (75.8)	2,026 (77.5)	113 (70.2)	139 (71.6)	91 (57.2)	<0.001
Smoking status baseline (%)						<0.001
Never	1,695 (54.2)	1,368 (52.4)	90 (55.9)	129 (66.5)	108 (67.9)	
Current	630 (20.1)	547 (20.9)	31 (19.3)	29 (14.9)	23 (14.5)	
Ex-smoker	802 (25.6)	698 (26.7)	40 (24.8)	36 (18.6)	28 (17.6)	
Mediterranean diet score [mean (SD)]	8.72 (2.83)	8.76 (2.84)	8.41 (2.69)	8.79 (2.71)	8.16 (2.81)	0.032
Energy kcal/day [mean (SD)]	2,437.81 (654.57)	2,447.51 (648.56)	2,434.14 (708.74)	2,380.77 (709.30)	2,351.64 (622.10)	0.186
Physical activity METs.min/day [mean (SD)]	310.56 (341.61)	316.44 (332.54)	278.69 (376.14)	291.62 (277.62)	269.29 (489.37)	0.165
Hypertension	1,318 (42.1)	1,087 (41.6)	58 (36.0)	91 (46.9)	82 (51.6)	0.023
Diabetes	339 (10.8)	273 (10.4)	15 (9.3)	28 (14.4)	23 (14.5)	0.061
Body weight variables						
BMI baseline, kg/m^2^ [mean (SD)]	27.29 (4.53)	27.22 (4.48)	27.52 (4.62)	27.24 (4.77)	28.33 (4.80)	0.024
BMI follow-up, kg/m^2^ [mean (SD)]	27.49 (4.56)	27.35 (4.48)	28.01 (4.78)	27.88 (4.77)	28.78 (5.22)	<0.001
BMI change, kg/m^2^ [mean (SD)]	0.19 (2.42)	0.13 (2.38)	0.49 (2.46)	0.64 (2.43)	0.45 (2.82)	0.005
Weight change, kg [mean (SD)]	0.53 (6.19)	0.39 (6.10)	1.29 (6.47)	1.39 (6.30)	1.01 (7.12)	0.035
Weight change, % [mean (SD)]	1.01 (7.88)	0.78 (7.56)	2.23 (8.84)	2.48 (9.57)	1.74 (9.42)	0.003
Weight gain >5% (%)	767 (24.5)	596 (22.8)	54 (33.5)	66 (34.0)	51 (32.1)	<0.001
Obese (BMI ≥ 30) baseline (%)	721 (23.1)	585 (22.4)	37 (23.0)	48 (24.7)	51 (32.1)	0.042
Obese (BMI ≥ 30) follow-up (%)	779 (24.9)	621 (23.8)	47 (29.2)	55 (28.4)	56 (35.2)	0.003
Depression variables						
PHQ-9 score at follow-up [mean (SD)]	3.06 (3.97)	2.62 (3.46)	5.07 (5.31)	5.46 (5.55)	5.33 (5.28)	<0.001
Depression during follow-up (%)	312 (10.0)	180 (6.9)	40 (24.8)	57 (29.4)	35 (22.0)	<0.001
Antidepressant use at baseline (%)	320 (10.2)	0 (0.0)	161 (100.0)	0 (0.0)	159 (100.0)	<0.001
Antidepressant use at follow-up (%)	353 (11.3)	0 (0.0)	0 (0.0)	194 (100.0)	159 (100.0)	<0.001

a p-Value for the ANOVA or chi-square test for the difference across the four trajectories of antidepressant use.

**Figure 1:**
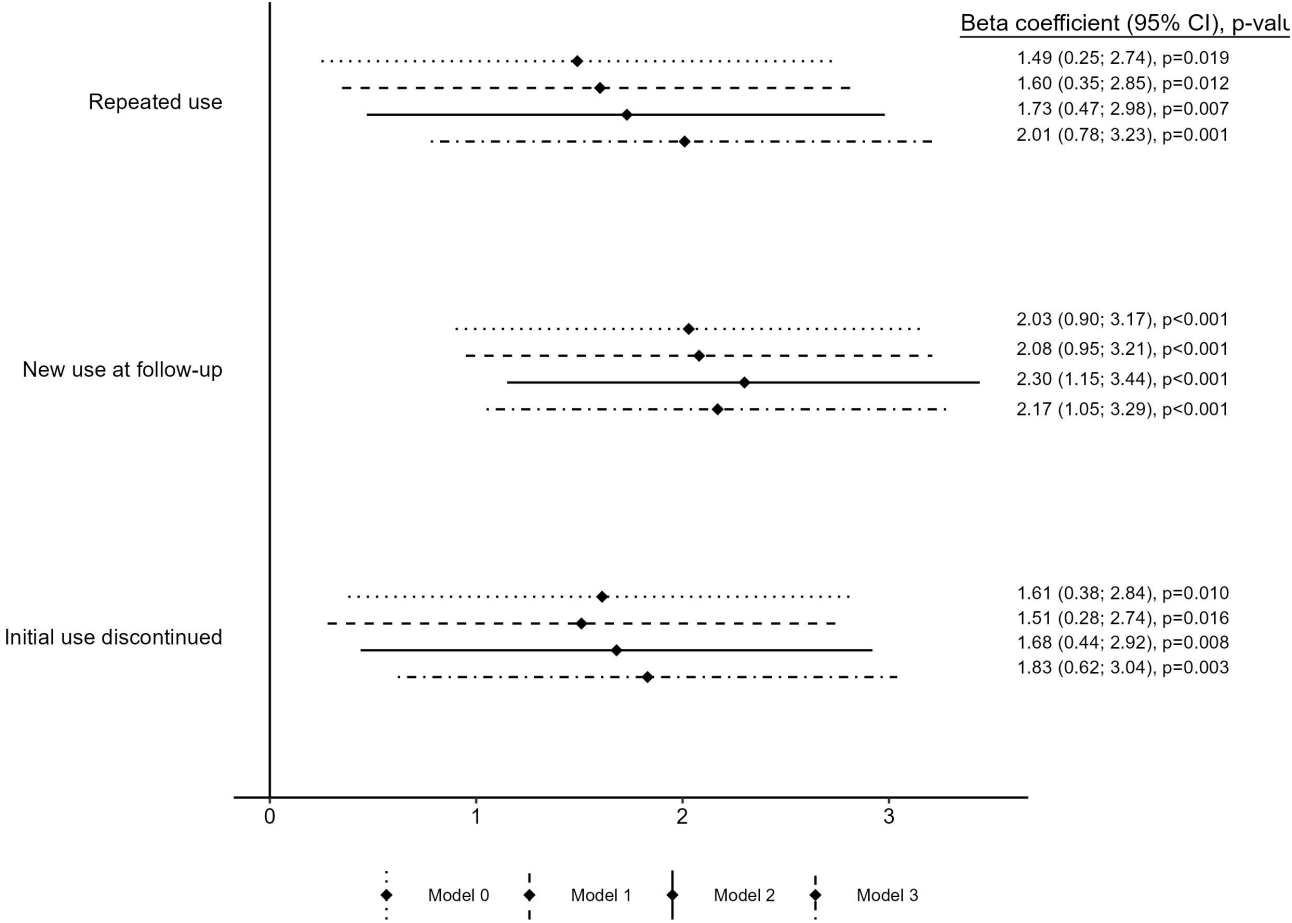
Linear association between antidepressant use trajectories and 6-year weight change expressed as percentage of baseline body weight. Model 0 includes baseline age and sex. Model 1 includes baseline age, sex, educational level, living with a partner, smoking, physical activity, Mediterranean diet adherence, diabetes, and hypertension. Model 2 further includes depression during follow-up. Model 3 further includes baseline BMI.

As seen in **Figure 1**, all three trajectories were associated with greater weight gain compared to never users of antidepressants at any level of adjustment for covariates. In Model 3, the greatest weight gain (change expressed as percent of baseline body weight) was observed for new users [+2.17% (1.05, 3.29%), p < 0.001] followed by repeated use [+2.01% (0.78, 3.23, p = 0.001)], and initial use discontinued [1.83% (0.62%, 3.04%), p = 0.003]. Adjustment for depression status during follow-up (Model 2) and baseline BMI (Model 3) did not attenuate the relationship; on the contrary, it was somewhat stronger for all three trajectories. Further adjustment for antipsychotics did not attenuate the coefficients either. When restricting to SSRI use (**Supplementary Table S3**), the associations were of similar magnitude except for repeated use, which displayed weaker weight gain than those of the other two trajectories.

**Figure 2:**
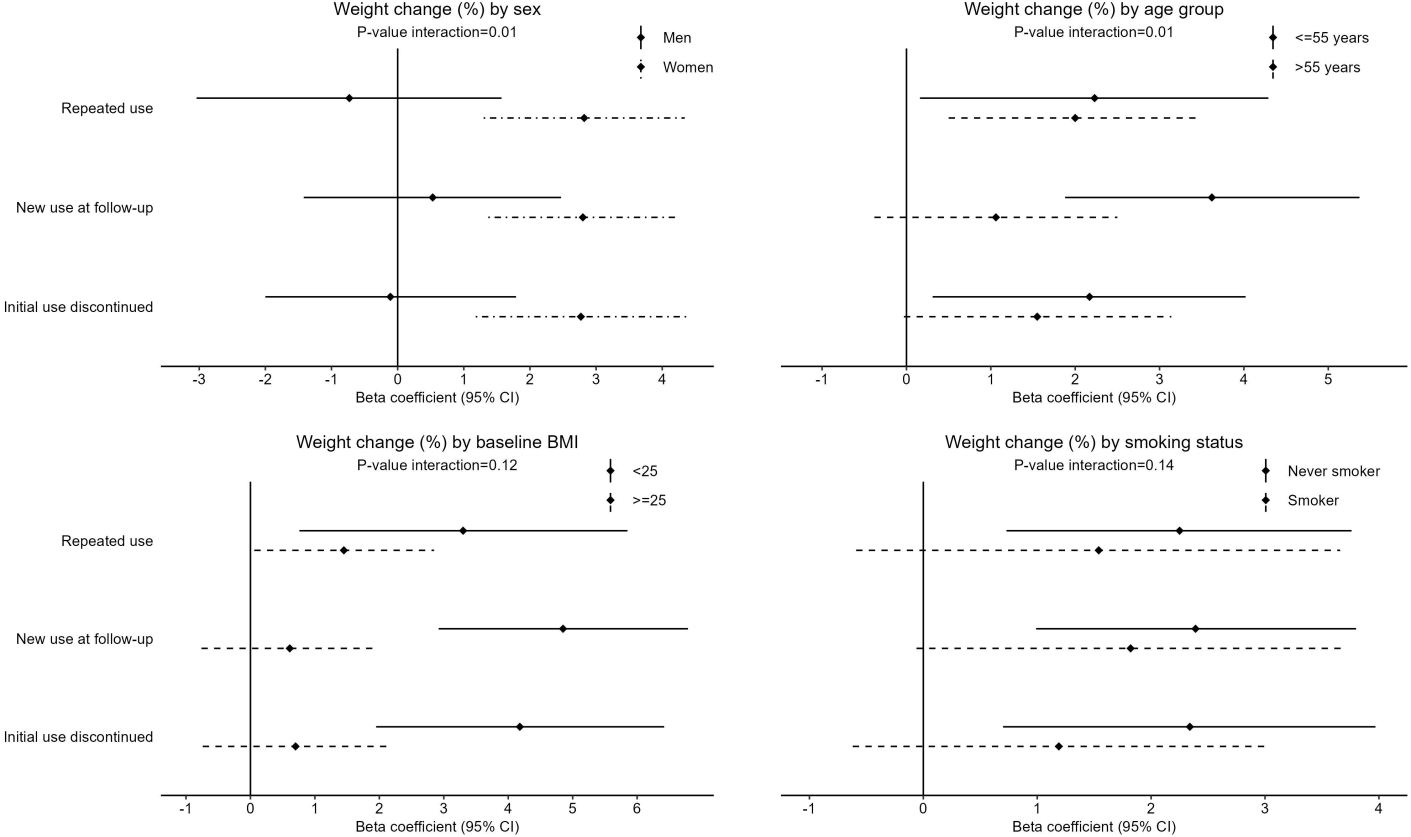
Subgroup analysis by sex, age, baseline BMI status and smoking status, for the linear association between antidepressant use trajectories and 6-year weight change, expressed as percentage of baseline body weight. Reference category is Never use of antidepressants. All coefficients are adjusted for (*when applicable*): baseline age, sex, educational level, living with a partner, smoking, physical activity, Mediterranean diet adherence, diabetes, hypertension, depression during follow-up, and baseline BMI (Model 3). p-Value for interaction from likelihood ratio test of nested models with and without interaction term between antidepressant use trajectory and sex (categorical), age (continuous), baseline BMI (continuous) and smoking status (categorical), respectively.

As seen in **Figure 2**, the associations were more pronounced in individuals younger than 55 years, in non-smokers, and in participants that were of normal weight (BMI < 25) (23) at baseline, and only apparent in women but not in men. Weight change over 6 years was different depending on baseline characteristics (**Supplementary Table S2**): only those participants with a baseline BMI <25 and younger than 55 years experienced weight gain. Women showed greater weight gain than men. Women and individuals aged >55 years were more likely to use antidepressants.

The results of the logistic regressions (**Supplementary Table S4**) revealed that being an ever user (at any time point) was associated with higher odds of >5% weight gain over 6 years, with ORs of very similar magnitude for all three trajectories: initial use discontinued was 1.80 (1.25, 2.56), for new use = 1.93 (1.38, 2.68), and for repeated use = 1.90 (1.31, 2.72).

**Figure 3:**
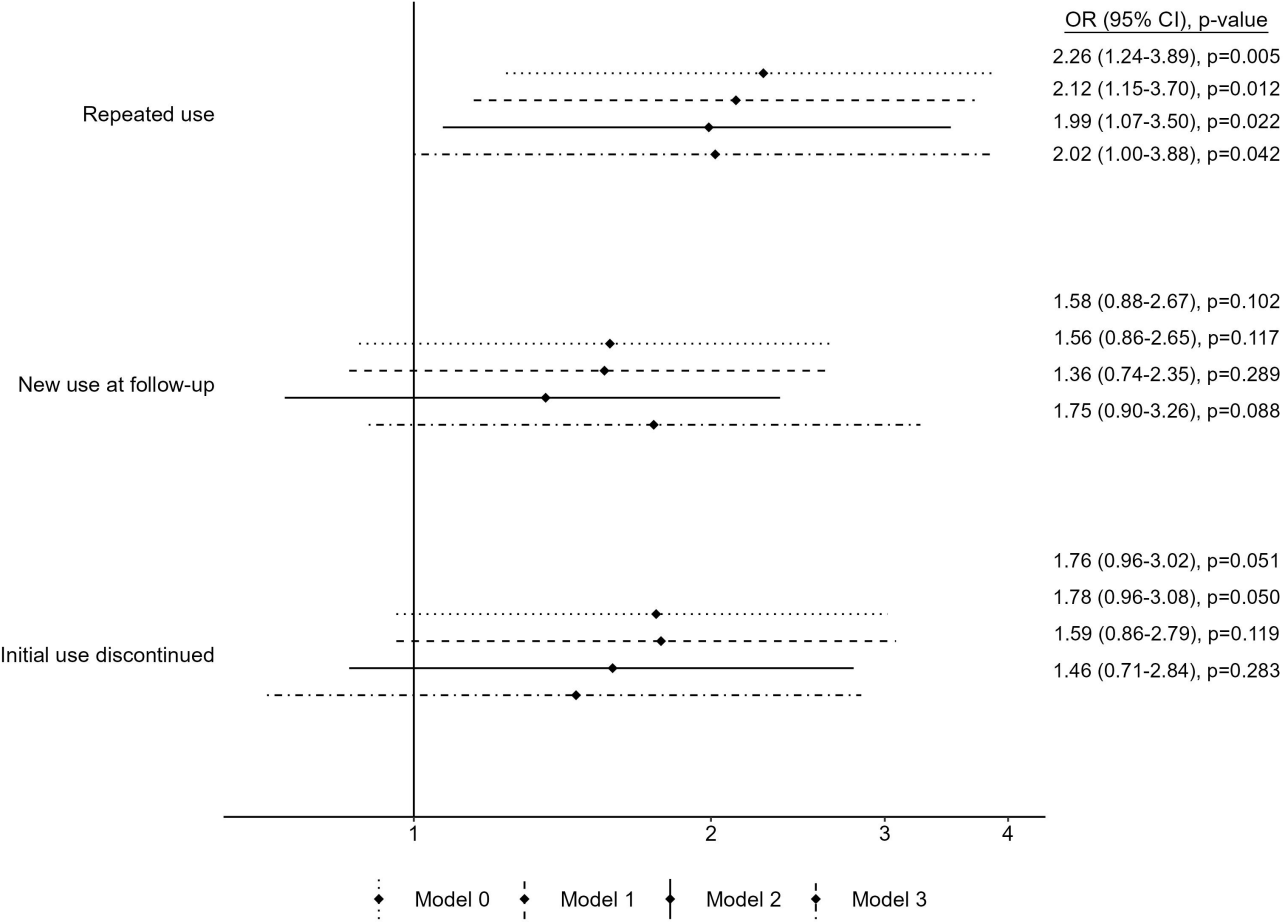
Association from multivariable logistic regressions between antidepressant use trajectories and odds of obesity in people without obesity at baseline (n = 2,404). Reference category is Never use of antidepressants. Model 0 includes baseline age and sex. Model 1 includes baseline age, sex, educational level, living with a partner, smoking, physical activity, Mediterranean diet adherence, diabetes, and hypertension. Model 2 further includes depression during follow-up. Model 3 further includes baseline BMI.

In participants without obesity at baseline (n = 2,404, **Figure 3**), only repeated use showed a strong and significant association with the odds for becoming obese: Model 3 OR = 2.02 (1.00, 3.88), while the other two trajectories presented ORs of lower magnitude and less precisely estimated. Moreover, depression was associated with higher odds of obesity [crude OR = 2.13 (1.40–3.17, p < 0.001), data not tabulated], and the addition of depression status in the models attenuated the associations between the trajectories of antidepressant use and obesity (Model 2 compared to Model 1).

## Discussion

In this population-based prospective cohort study of over 3,000 Spanish middle-aged adults, we found that the use of antidepressants, even if not sustained over time, was associated with greater weight gain over 6 years, ranging 1.78% to 2.08% greater increase in body weight increase compared with never use of antidepressant. This was independent of age, sex, socioeconomic and lifestyle factors, and depressive symptoms. The association was most pronounced in women, individuals aged under 55 years of age, and those with a healthy body weight at baseline. Among participants without obesity at baseline, repeated use of antidepressants (both at baseline and 6 years later) was associated with higher risk of becoming obese.

Our findings contribute to the growing body of evidence, including studies from the UK (18), Finland (19), Canada (20, 31), Australia (32), and Netherlands (21), which show that overall antidepressant use, particularly SSRIs and potentially TCAs over the long term, is associated with greater weight gain. The magnitude of weight gain we observed is comparable to, or greater than, what has been reported in other studies on antidepressant use versus no use. It has been suggested that these observations may relate to depression rather than use of medication, but our results, alongside others (18, 19, 21, 31, 32), adjust for depressive symptoms, and this does not attenuate the associations between antidepressant use and weight gain. However, it is important to note that we only measured depressive symptoms at follow-up. In the “new use” group, participants who gained weight might have had more depressive symptoms at baseline and started antidepressant treatment soon after, but we cannot fully disentangle this with the available data. The strong association in this group may also indicate that antidepressants have a more pronounced short-term impact on weight gain. Long-term antidepressant users started the study with a higher baseline body weight (BMI: 28.3) compared to both never users and new users at follow-up (BMI: 27.2) suggesting that weight gain linked to antidepressant use may have occurred before the study began. Additionally, a higher initial body weight limits the potential for further weight gain over time.

Regarding subgroup analyses, our results showing that the association was only apparent in women are consistent with one previous study (21), although other studies found no significant sex differences (18, 31). It is important to note that 6-year weight gain was on average higher and with a greater variability in women than in men, and the proportion of antidepressant users was also higher, which can explain why the associations with antidepressant were stronger in women. The stronger association between new antidepressant use and weight gain observed in younger individuals and in those with a healthier BMI has also been observed by Gafoor et al. (18) and can also be explained by a greater weight gain observed in the younger group. Given that the onset of depression typically occurs in young adulthood, this underscores the importance of carefully managing weight when prescribing antidepressants to younger adults, even though the youngest participants in our study were aged 35.

We found that for participants without obesity, the risk of becoming obese was increased mostly for people with a repeated use of antidepressants and that discontinuing or initiating antidepressants was not clearly associated with obesity risk. This implies that particular care should be given in weight monitoring of long-term users of antidepressants to prevent transitioning to obesity.

Several mechanisms could explain the observed associations, including appetite hormones, eating behaviors, and metabolic pathways that are regulated by monoamine pathways (serotonergic, adrenergic, dopaminergic, histaminergic, and muscarinic receptors) (8). For instance, the histaminergic system is involved in appetite control, and the anti-histaminergic effects of some antidepressants can result in increased appetite by activating orexigenic neuropeptides like ghrelin and NPY. In the case of SSRIs, this drug class inhibits serotonin transporter (SERT) function, and SERT deficiency has been linked to obesity and diabetes in mice (33).

Our study has a several strengths. It is a population-based cohort of reasonably large sample size aiming to be representative of adults living in the province of Girona, in northeast Spain. The use of antidepressants was frequent (16.4%) and comparable to data from other high-income countries such as the UK (18%) (18) or Canada (13.7%) (31). The follow-up period was also long enough to capture long-term associations, in contrast to most clinical trials focusing on short- (weeks) and medium-term (approximately 6 months) side effects (14). Furthermore, both exposure and outcome were well characterized: body weight and height were measured using standard methods by trained nurses, and use of medication was reported during visits and verified by the attending nurse. This contrasts with other long-term observational studies that rely on clinical health records where anthropometric information is heterogeneously reported (18), or other cohorts where it is self-reported (19, 20), and rely on prescription, which may not reflect actual use by the patients (18, 19, 32). A unique feature of our study is the detailed characterization of lifestyle factors (physical activity, diet, and smoking), which likely affect adiposity and could confound the association with antidepressants, factors that have only been considered in one previous study (32).

However, there are limitations. First, while prescription data are more objective and frequently updated, our study relied on self-reported drug use at only two time points. Additionally, the re-classification of medication at baseline and at follow-up differed slightly (no TCA at follow-up) likely due to the shift from TCAs to SSRIs or NDRIs during this period (18). Another limitation is that we could not assess trajectory of different subclasses of antidepressants and had to treat “any antidepressant” use as the main exposure. The effect on weight gain likely varies between drug classes, with TCAs generally associated with greater weight gain, although recent long-term studies suggest a clearer association between SSRIs and weight gain (18, 20, 21, 32), which we also observed in our sensitivity analysis restricting to SSRIs. The lack of detailed information on specific drugs is a limitation of our study, especially given recent evidence showing differential weight gain by antidepressant class (15). Moreover, due to the low number of reported antipsychotic use, we could not assess properly the association of dual use of antidepressant and antipsychotic medications. However, when adjusting for antipsychotic use, associations with antidepressant use remained unchanged suggesting weak confounding in our study. Additionally, there may be classification bias, where some participants classified as repeated users may not have used antidepressants consistently between visits, or “never users” may have used antidepressants between visits without reporting it. As with any observational study, residual confounding and potential collider bias are concerns. For instance, follow-up participation could be linked to both antidepressant use and weight gain. However, despite differences in the profiles of participants who did not attend the follow-up, their baseline BMI and antidepressant use did not differ significantly from those who completed the follow-up.

To address these limitations, future research should not only focus on antidepressant but other psychoactive drugs such as antipsychotics. Ideally, future studies should combine cohort data with electronic health records to increase sample size and follow-up time, capture key lifestyle confounders and prescription trajectories, account for concomitant medications that affect body weight, and focus on younger populations.

To conclude, in this population-based study of Spanish adults, compared with never use over 6 years, any use of antidepressant, even discontinued, was associated with greater weight gain or risk of gaining >5% of body weight over 6 years. Repeated use was also associated with greater risk of developing obesity for people without obesity at baseline. Given the global obesity epidemic and the widespread use of antidepressants, weight management and metabolic monitoring should be encouraged and integrated into depression follow-up guidelines alongside antidepressant prescriptions.

## Data Availability

The raw data supporting the conclusions of this article will be made available by the authors, without undue reservation.
