# Supplementary material for: Trajectories of antidepressant use and 6-year change in body weight: a prospective population-based cohort study
Source: Front Psychiatry. 2024 Dec 24;15:1464898. doi: 10.3389/fpsyt.2024.1464898 (PMC11703859; doi:10.3389/fpsyt.2024.1464898)

Table S1. Comparison between participants included in the analyses and those excluded

	Included	Excluded	p-value
N	3127	3225	
Female (%)	1701 (54.4)	1628 (50.5)	0.002
Age (mean (SD))	55.56 (11.61)	57.76 (13.38)	<0.001
Educational level (%)			0.001
High	715 (22.9)	676 (21.5)	
Medium	926 (29.6)	831 (26.4)	
Low	1486 (47.5)	1639 (52.1)	
Smoking status baseline (%)			<0.001
Never	1695 (54.2)	1524 (48.2)	
Current	630 (20.1)	763 (24.1)	
Ex-smoker	802 (25.6)	876 (27.7)	
Mediterranean diet score (mean (SD))	8.72 (2.83)	8.70 (2.76)	0.857
Physical activity METs.min/day (mean (SD))	310.56 (341.61)	317.61 (322.67)	0.398
BMI baseline; kg/m2 (mean (SD))	27.29 (4.53)	27.42 (4.64)	0.268
Obese (BMI>=30) baseline = 1 (%)	721 (23.1)	819 (25.8)	0.014
Antidepressant use at baseline (%)	320 (10.2)	370 (11.5)	0.114

Table S2. Descriptive characteristics by baseline age, sex, and baseline BMI

	By baseline age			By sex			By baseline BMI		
	<55y	>=55y	p-value	Male	Female	p-value	<25 kg/m2	>=25 kg/m2	p-value
n (%)	1494	1633		1426	1701		991	2134	
Female (%)	840 (56.2)	861 (52.7)	0.054	0 (0.0)	1701 (100.0)	<0.001	687 (69.3)	1013 (47.5)	<0.001
Age (mean (SD))	45.36 (5.48)	64.88 (6.96)	<0.001	56.05 (11.57)	55.14 (11.63)	0.029	51.91 (11.41)	57.24 (11.31)	<0.001
Educational level (%)			<0.001			0.019			<0.001
High	458 (30.7)	257 (15.7)		359 (25.2)	356 (20.9)		320 (32.3)	395 (18.5)	
Low	478 (32.0)	1008 (61.7)		655 (45.9)	831 (48.9)		325 (32.8)	1159 (54.3)	
Medium	558 (37.3)	368 (22.5)		412 (28.9)	514 (30.2)		346 (34.9)	580 (27.2)	
Living with a partner (%)	1206 (80.7)	1163 (71.2)	<0.001	1217 (85.3)	1152 (67.7)	<0.001	739 (74.6)	1629 (76.3)	0.305
Smoking status baseline (%)			<0.001			<0.001			<0.001
Never	673 (45.0)	1022 (62.6)		473 (33.2)	1222 (71.8)		522 (52.7)	1172 (54.9)	
Current	432 (28.9)	198 (12.1)		384 (26.9)	246 (14.5)		240 (24.2)	390 (18.3)	
Ex-smoker	389 (26.0)	413 (25.3)		569 (39.9)	233 (13.7)		229 (23.1)	572 (26.8)	
Mediterranean diet score (mean (SD))	8.08 (2.83)	9.29 (2.70)	<0.001	8.98 (2.91)	8.49 (2.74)	<0.001	8.59 (2.86)	8.77 (2.81)	0.087
Energy kcal/day (mean (SD))	2558 (649)	2328 (640)	<0.001	2486 (675)	2398 (634)	<0.001	2493 (654)	2412 (654)	0.001
Physical activity METs.min/day (mean (SD))	277.0 (279.0)	341.2 (387.8)	<0.001	374.7 (395.2)	256.8 (278.0)	<0.001	303.8 (322.0)	313.4 (350.4)	0.464
Hypertension	374 (25.0)	944 (57.8)	<0.001	669 (46.9)	649 (38.2)	<0.001	221 (22.3)	1096 (51.4)	<0.001
Diabetes	77 (5.2)	262 (16.0)	<0.001	191 (13.4)	148 (8.7)	<0.001	50 (5.0)	289 (13.5)	<0.001
**Body weight variables**									
BMI baseline, kg/m2 (mean (SD))	26.44 (4.57)	28.08 (4.34)	<0.001	27.81 (3.89)	26.86 (4.96)	<0.001	22.66 (1.74)	29.45 (3.74)	<0.001
BMI follow-up, kg/m2 (mean (SD))	26.91 (4.66)	28.01 (4.41)	<0.001	27.88 (3.87)	27.16 (5.05)	<0.001	23.36 (2.39)	29.41 (4.03)	<0.001
BMI change, kg/m2 (mean (SD))	0.48 (2.42)	-0.07 (2.39)	<0.001	0.07 (2.19)	0.30 (2.59)	0.008	0.69 (1.86)	-0.04 (2.60)	<0.001
Weight change, kg (mean (SD))	1.48 (6.34)	-0.35 (5.91)	<0.001	0.40 (6.02)	0.64 (6.33)	0.279	1.86 (4.82)	-0.09 (6.64)	<0.001
Weight change, % (mean (SD))	2.40 (8.03)	-0.27 (7.52)	<0.001	0.71 (6.83)	1.26 (8.66)	0.054	3.16 (8.06)	0.01 (7.60)	<0.001
Weight gain >5% (%)	466 (31.2)	301 (18.4)	<0.001	306 (21.5)	461 (27.1)	<0.001	330 (33.3)	437 (20.5)	<0.001
Obese (BMI>=30) baseline (%)	274 (18.3)	447 (27.4)	<0.001	338 (23.7)	383 (22.5)	0.728	0 (0.0)	721 (33.8)	
Obese (BMI>=30) follow-up (%)	318 (21.3)	461 (28.2)	<0.001	356 (25.0)	423 (24.9)	0.983	9 (0.9)	770 (36.1)	<0.001

**Depression variables**									
PHQ-9 score at follow-up (mean (SD))	3.05 (3.98)	3.06 (3.97)	0.928	2.03 (2.92)	3.92 (4.50)	<0.001	3.00 (3.79)	3.08 (4.06)	0.583
Depression during follow-up (%)	114 (7.6)	118 (7.2)	0.717	48 (3.4)	184 (10.8)	<0.001	67 (6.8)	165 (7.7)	0.373
Antidepressant use at baseline (%)	119 (8.0)	201 (12.3)	<0.001	80 (5.6)	240 (14.1)	<0.001	89 (9.0)	231 (10.8)	0.129
Antidepressant use at follow-up (%)	129 (8.6)	224 (13.7)	<0.001	78 (5.5)	275 (16.2)	<0.001	109 (11.0)	244 (11.4)	0.767

Table S3. Association from linear regressions between SSRI use trajectories and 6-year weight change expressed as percentage of baseline body weight

	SSRI use			
Adjustment	Never use [a]	Initial use discontinued [a]	New use at follow-up [a]	Repeated use [a]
N	2960	162	207	127
Model 0 [b]	Reference	2.23 (0.92 to 3.54, p=0.001)	2.03 (0.87 to 3.18, p=0.001)	0.96 (-0.48 to 2.40, p=0.193)
Model 1 [c]	Reference	2.16 (0.86 to 3.47, p=0.001)	2.09 (0.93 to 3.24, p<0.001)	1.08 (-0.36 to 2.53, p=0.141)
Model 2 [d]	Reference	2.33 (1.01 to 3.64, p=0.001)	2.31 (1.14 to 3.47, p<0.001)	1.23 (-0.22 to 2.68, p=0.096)
Model 3 [e]	Reference	2.44 (1.16 to 3.72, p<0.001)	2.22 (1.08 to 3.37, p<0.001)	1.58 (0.16 to 2.99, p=0.029)

[a] Values are beta coefficients (95% confidence interval; p-value)

[b] Model 0 includes baseline age and sex

[c] Model 1 includes baseline age; sex; educational level; living with a partner; smoking; physical activity; Mediterranean diet adherence; diabetes and hypertension

[d] Model 2 further includes depression during follow-up

[e] Model 3 further includes baseline BMI

Table S4. Association from multivariable logistic regressions between antidepressant use trajectories and odds of weight gain >5% of baseline body weight

Adjustment	Never use [a]	Initial use discontinued [a]	New use at follow-up [a]	Repeated use [a]
N cases / N tot	596/2613	54/161	66/194	51/159
Model 0 [b]	1.00 (Reference)	1.78 (1.25-2.52, p=0.001)	1.89 (1.37-2.60, p<0.001)	1.83 (1.27-2.60, p=0.001)
Model 1 [c]	1.00 (Reference)	1.72 (1.20-2.43, p=0.002)	1.90 (1.37-2.62, p<0.001)	1.79 (1.23-2.56, p=0.002)
Model 2 [d]	1.00 (Reference)	1.76 (1.23-2.50, p=0.001)	1.96 (1.40-2.71, p<0.001)	1.82 (1.25-2.61, p=0.001)
Model 3 [e]	1.00 (Reference)	1.80 (1.25-2.56, p=0.001)	1.93 (1.38-2.68, p<0.001)	1.90 (1.31-2.72, p=0.001)

[a] Values are odds ratios (95% confidence interval; p-value)
[b] Model 0 includes baseline age and sex
[c] Model 1 includes baseline age; sex; educational level; living with a partner; smoking; physical activity; Mediterranean diet adherence; diabetes and hypertension
[d] Model 2 further includes depression during follow-up
[e] Model 3 further includes baseline BMI

Figure S1. Flow chart of the selection of study participants included in the analysis

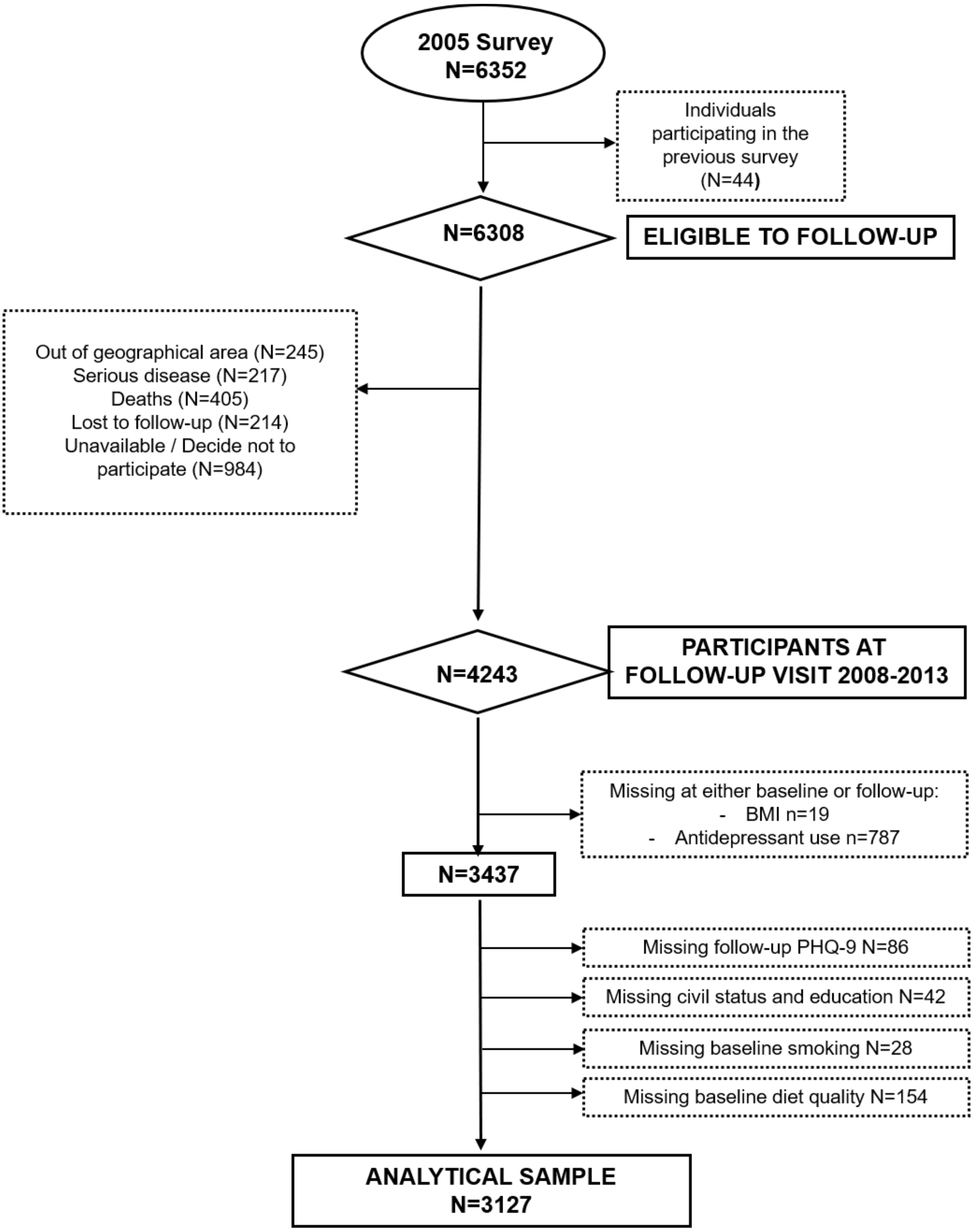

Supplement: Supplementary file 1 [file DataSheet1.pdf]
